# An Independent Psychometric Evaluation of the PROMS Measure of Music Perception Skills

**DOI:** 10.1371/journal.pone.0159103

**Published:** 2016-07-11

**Authors:** Richard Kunert, Roel M. Willems, Peter Hagoort

**Affiliations:** 1 Max Planck Institute for Psycholinguistics, Nijmegen, The Netherlands; 2 Radboud University Nijmegen, Donders Institute for Brain, Cognition and Behavior, Nijmegen, The Netherlands; 3 Radboud University Nijmegen, Centre for Language Studies, Nijmegen, The Netherlands; University of Zurich, SWITZERLAND

## Abstract

The Profile of Music Perception Skills (PROMS) is a recently developed measure of perceptual music skills which has been shown to have promising psychometric properties. In this paper we extend the evaluation of its brief version to three kinds of validity using an individual difference approach. The brief PROMS displays good discriminant validity with working memory, given that it does not correlate with backward digit span (*r* = .04). Moreover, it shows promising criterion validity (association with musical training (*r* = .45), musicianship status (*r* = .48), and self-rated musical talent (*r* = .51)). Finally, its convergent validity, i.e. relation to an unrelated measure of music perception skills, was assessed by correlating the brief PROMS to harmonic closure judgment accuracy. Two independent samples point to good convergent validity of the brief PROMS (*r* = .36; *r* = .40). The same association is still significant in one of the samples when including self-reported music skill in a partial correlation (*r*_partial_ = .30; *r*_partial_ = .17). Overall, the results show that the brief version of the PROMS displays a very good pattern of construct validity. Especially its tuning subtest stands out as a valuable part for music skill evaluations in Western samples. We conclude by briefly discussing the choice faced by music cognition researchers between different musical aptitude measures of which the brief PROMS is a well evaluated example.

## Introduction

Perceptual music skills differ widely in the population: from amusic individuals who exhibit impaired music listening skills [1] to highly proficient people scoring highly on musical skill measures [2]. There is growing interest in these inter-individual differences, partly because evidence is accumulating that musical and non-musical faculties are related. For example, music skills have been linked to native and non-native language abilities [3,4].

However, progress in music cognition has been hampered by an absence of modern, objective measurement tools which are both fast as well as easy to administer and psychometrically validated. A variety of novel musical skill measures has been proposed to fill this gap [5–9]. This publication is concerned with the psychometric evaluation of one measure for perceptual music skills (the profile of music perception skills; PROMS [2]; www.zentnerlab.com/psychological-tests/the-profile-of-music-perception-skills) which has already been adopted by researchers interested in music cognition [10].

As most other music skill measures the PROMS requires participants to judge whether a reference and a probe stimulus are the same or not. The comparison can be performed based on different music features such as melody, rhythm, or tuning. While the full PROMS is based on nine subtests assessing a different music feature each, the brief version—which we focus on here—comprises only four (comparison of stimuli based on melody, tuning, tempo, or rhythmic accent) taking about half an hour to administer. The administration time is somewhat higher than that of other novel musical skill measures aimed at adults (11 minutes for SMDT [6]; 18 minutes for the MET [8]; 20–25 minutes for the Gold-MSI [11]). However, in return, the brief PROMS provides more subtests (4 for PROMS; 2 for MET; 3 for SMDT; Gold-MSI includes 2 music measures according to [11] and 4 according to the website http://www.gold.ac.uk/music-mind-brain/gold-msi/) allowing for a more fine-grained assessment of music skills and subskills.

This raises the question of what the PROMS actually purports to assess. We define the measured concept—music perception skills—along the same lines as [2]. The focus lies on rather elementary aspects of music which can be found across musical systems and traditions, such as the use of discrete pitch, tempo, precise rhythms and melodic lines [12]. While this does not render the PROMS culture-free, it allows for insights into musicality which are potentially wider ranging than the Western cultural context. We are uncommitted as to the origin of musical skills, whether they are due to deliberate practice [13] or talent/giftedness [14]. The advantage of musical skill measures such as the PROMS is that they can be used to answer questions regarding the origins of musical skills instead of relying on skill operationalisations (e.g., musical instrument proficiency) which require musical expertise. Thus, the PROMS aims to measure basic music abilities in the general population, including musically trained and untrained individuals.

Whether the PROMS achieves its aim of measuring music perception skills has been assessed psychometrically. Law and Zentner [2] reported high internal consistency as well as good test-retest reliability for the brief PROMS. Furthermore, the full PROMS, with which the brief version correlates at *r* = .95, has been shown to have convergent validity with other measures of music ability, i.e. it appears to measure the same concept as established music ability tests. Also, it exhibits criterion validity with various measures of music achievement, i.e. it is related to ‘real world’ variables of musical skill such as musical training and musicianship status. Furthermore, Law and Zentner [2] investigated its discriminant validity, i.e. whether the PROMS is *not* associated with a task measuring an unrelated concept. The unrelated task was a gap detection task in which participants had to detect short gaps of silence in white noise. Given that none of the correlations between sub-test scores and gap-detection performance reaches significance, performance on the PROMS cannot be equated with nonmusical auditory discrimination abilities.

While these results impressively demonstrate the good test properties of the PROMS, open questions remain. First of all, Law and Zentner [2] established discriminant validity solely with a gap-detection task even though it is known that similar music skill measures suffer from a working memory confound. That is to say that holding a reference stimulus in mind in order to compare it to a probe stimulus requires working memory resources whose efficiency and size can consequently influence test scores, as seen for similar music ability measures (e.g., [3,8,15]). Therefore, we evaluate the relation of the brief PROMS to a standard measure of working memory: digit span. Its forward subscore is usually thought to measure short-term memory (store information temporarily) while its backward subscore is related to working memory (holding information online for active processing).

Secondly, we sought to replicate and extend Law and Zentner’s [2] assessment of criterion validity by comparing brief PROMS scores to values measuring musical training (years of training, musicianship status) as before. However, we also include self-reported musical talent in this assessment in order to evaluate whether musical skill as measured by the PROMS is related to musical talent as commonly understood in the general population.

Thirdly, convergent validity of the PROMS has so far only been established through a comparison with other music ability measures which are, crucially, also based on the judged similarity of a reference and a comparison stimulus. Therefore, we investigate the test’s convergent validity by comparing it to a different kind of task. This new task is based on a music feature (harmony) which is not directly measured by any of the subtests of the brief PROMS. Furthermore, the task (closure ratings) is different in kind to the usual music ability measures as it does not rely on an auditory discrimination between two stimuli. Therefore, convergent validity aims to see whether the assessment of perceptive music skills in one task (PROMS) is related to the same assessment in a completely different task (harmony judgments).

A music ability measure with good psychometric properties is essential in order to investigate the influence of music skills on cognition as well as the underlying reason for why some people are ‘good at music’. Therefore, it is important to rigorously assess newly developed measures of music ability before they are widely adopted. The current investigation does just that for the brief PROMS.

## Methods

### Ethics Statement

Written informed consent was obtained from all participants prior to measurement and the study received ethical approval from the local reviewing committee ‘‘CMO Arnhem Nijmegen” (CMO no 2001/095 and amendment ‘‘Imaging Human Cognition” 2006, 2008), in accordance with the Research involving human subjects Act, following the principles of the Declaration of Helsinki.

### Participants

Our data are based on main task, pretest, post-test, and pilot participant data principally acquired for an independent research question [16]. The full sample consists of 161 Dutch participants (37 males) aged 18 to 64 (*M* = 22.80; *SD* = 4.75), with little formal musical training (*M* = 4.39 years; min = 0; max = 20; *SD* = 4.46). Most participants (54%) described themselves as non-musicians, 39% as amateur and 7% as semi-professional musicians. 14% reported being left-handed. All were paid for their participation or received undergraduate course credit. Not all participants took part in all aspects of the study. Therefore, the number of people available for specific analyses differs between 53 and 160 (see sample sizes per analysis in brackets below).

### Tasks

The brief PROMS was administered in the lab as a web-based test at the end of a testing session. The brief PROMS assesses melody first, followed by tuning, tempo, and rhythmic accent. Participants are asked to judge whether a standard stimulus, which is repeated, is identical to a comparison stimulus. Answers are given on a five-point Likert scale providing a coarse measure of confidence ("definitely same", "probably same", "I don’t know", "probably different", and "definitely different"). Each subtest includes 18 trials.

In the melody subtest participants hear a two-bar monophonic harpsichord melody twice, followed by the probe melody which can differ slightly by one or more tones. The tuning subtest plays a C-chord whose tone E could be mistuned. Participants are asked to judge whether the tuning is the same in the reference and the probe stimulus. The tempo subtest comprises rhythmically and timbrally diverse stimuli which are the same between reference and probe stimulus except, potentially, for their tempo. Finally, the rhythmic accent subtest uses non-melodic rhythmic sequences played with a rim-shot timbre. Participants are asked to detect whether intensity accents are placed on the same notes in the reference stimulus and the probe stimulus.

In order to measure working memory, digit span was acquired in Dutch. The task contains only single digits which are read out by the experimenter (RK) at a rate of approximately one per second. Participants were tested individually. The experimenter asked them to repeat progressively longer sequences of digits in the same order (forward digit span) or in reverse order (backward digit span) until they failed on two trials of the same length [17].

**Fig 1:**
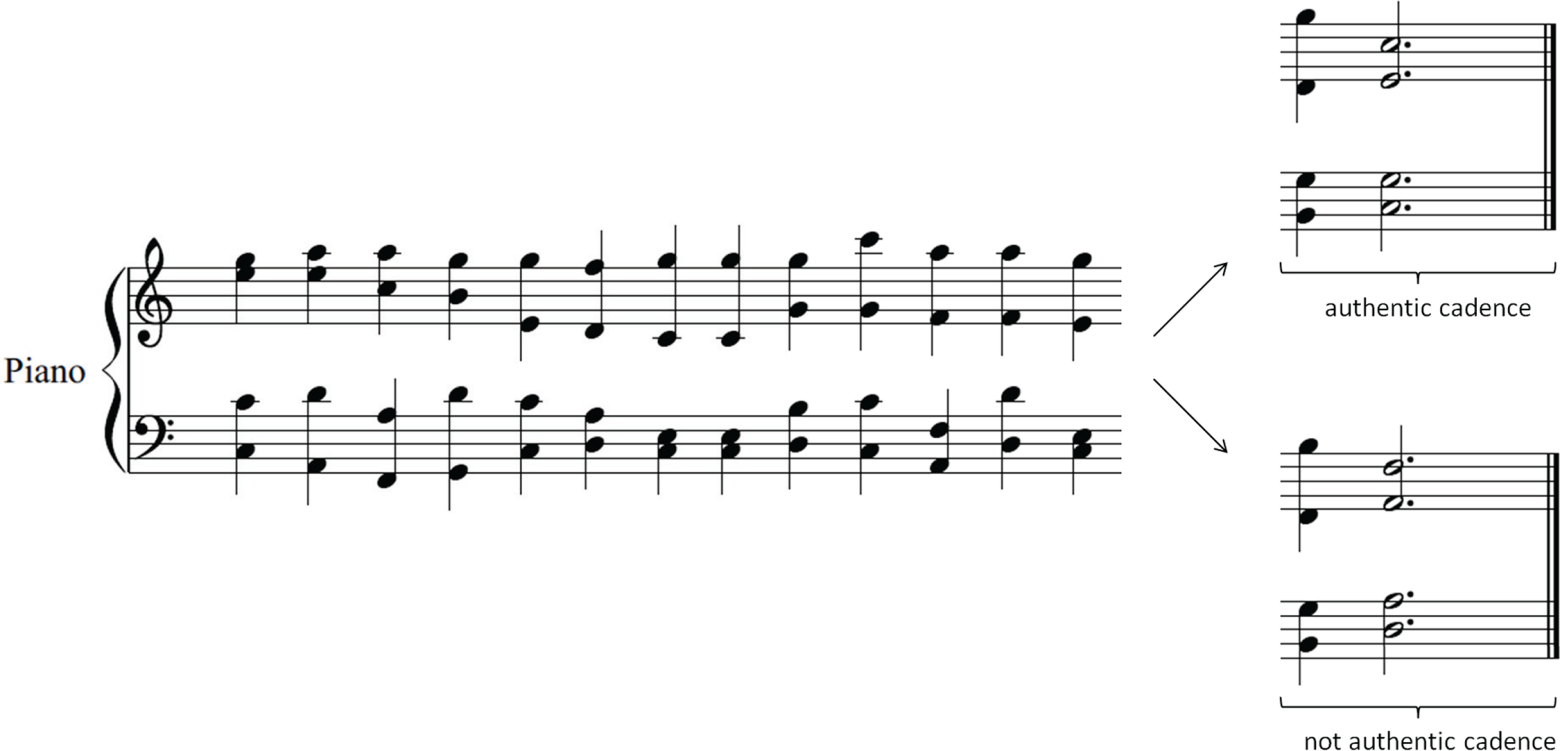
Musical task item. Participants are required to rate the closure (feeling of completion) of chord progressions ending either on an authentic cadence (top ending) or not (bottom ending), i.e. a dominant followed by a supertonic (shown here) or followed by a subdominant (not shown). Accuracy refers to the average rating of sequences ending on an authentic cadence minus the average rating of no cadence endings.

The closure rating task, which we use in order to measure musical task accuracy, is based on ten harmonic sequences made up of 14 chords played with a piano timbre at 96 bpm; see Fig 1. Participants are asked to judge their feeling of completeness, i.e. to what extent they feel the music stimulus has ended instead of being cut early (seven point Likert scale). Unbeknownst to the participants, sequences end either on an authentic cadence (dominant followed by tonic) or not (dominant followed by supertonic or subdominant). The former usually result in a high closure rating, the latter in a low closure rating. Original sequences are transposed twice resulting in 60 harmonic sequences (10 items × 2 endings × 3 transpositions).

60 participants were required to perform the music task in isolation (post-test participants in [16]). Their data are based on all 40 trials (20 ending on authentic cadence, 20 not) which are entered into the analysis under the label ‘full attention’. Given the moderate sample size, we sought to replicate the findings of this sample with a new set of 56 other participants performing the music task while simultaneously solving a reading task (one word per chord presented visually) or an arithmetic task (one number or operator per chord presented visually) (experiment 1 participants in [16]). The trials analyzed here constituted the filler trials in the original study, i.e. their language/arithmetic dimension was variable but relatively easy. All 100 filler trials (50 ending on authentic cadence, 50 not) entered into the analysis labeled as ‘divided attention’.

Our musical task accuracy measure is novel, requiring some basic validation. Regarding its internal consistency, Cronbach’s α is .90 overall (117 participants, 10 items). This suggests good to excellent internal consistency. Regarding its external validity, our musical task accuracy measure does correlate with the number of years of formal musical training (*r*_(117)_ = .21, *p* = .02). This suggests that it is related to at least one real-world measure of musical skill.

### Analysis

Raw data and analysis code written in R are available as supplementary information (S1 File, S2 File, S3 File and S4 File). The brief PROMS scores were derived from the web-based feedback screen. Per trial, participants receive 1 point for a correct response chosen with maximum confidence, half a point for a correct response chosen with less confidence, and zero points for an incorrect or ‘I don’t know’ response. The maximum possible score, therefore is 18 points per subtest and 72 points overall. For forward and backward digit span we used the number of correct trials. For the closure rating task we derived a difference score from the ratings (authentic cadence minus no-cadence).

In order to check whether the correlations we report are robust, we compare the reported Pearson correlation values to Spearman rank order correlations and to iterated re-weighted least squares regressions which weigh down data points with large residuals. The results are very similar for all three methods.

**Fig 2:**
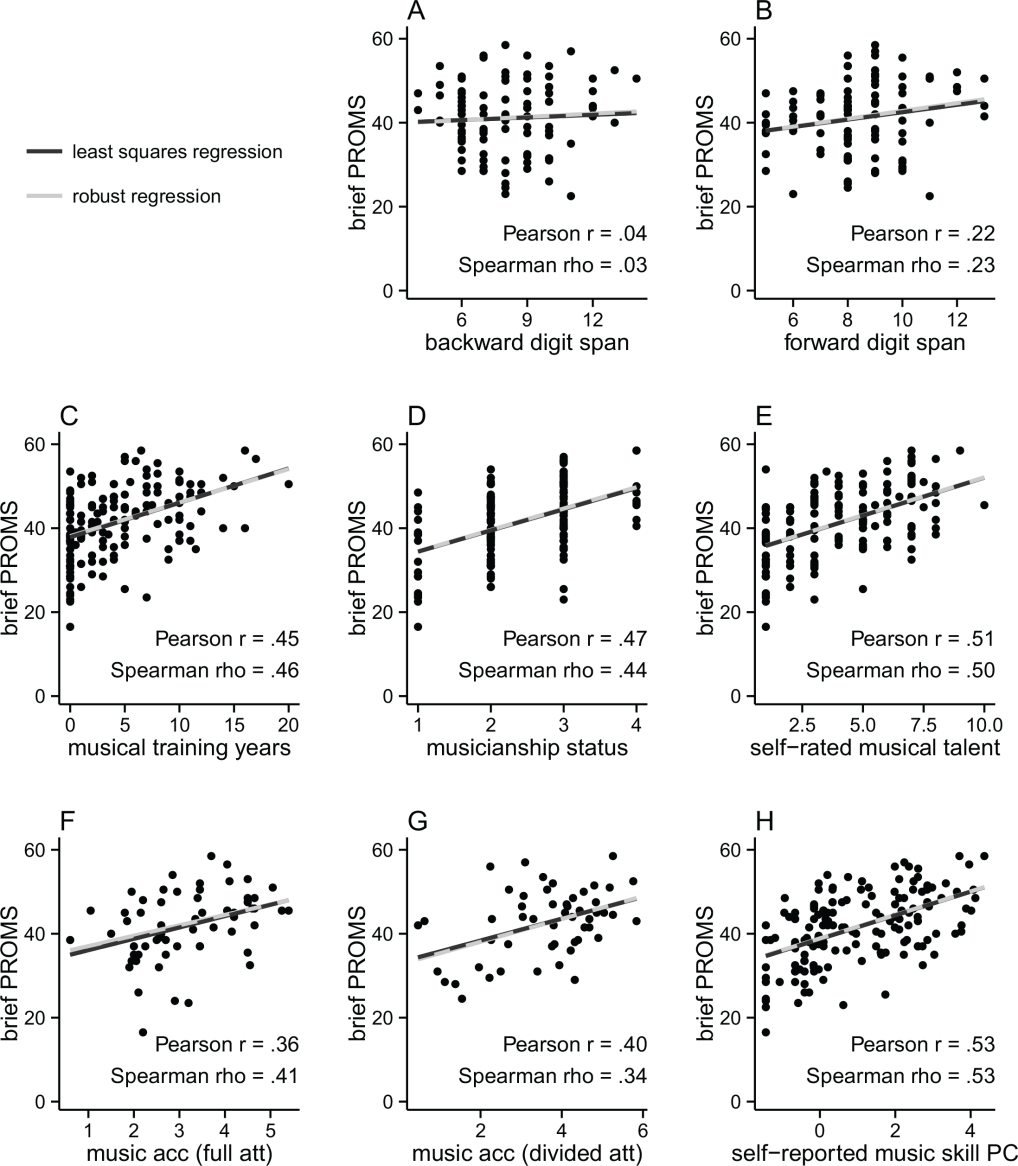
Correlation of brief PROMS total scores with validity measures. Two lines are fitted to the data, a linear fit (dark) corresponding to Pearson *r* and a robust fit (light) corresponding to an iterated re-weighted least squares regression. Overlapping lines are plotted as a dashed dark-light line. For inferential tests and PROMS subtest scores, see Table 3. music acc (full att) = musical task accuracy (full attention); music acc (divided att) = musical task accuracy (divided attention); self-reported music skill PC = first principal component combining musical training years, musicianship status, and self-rated musical talent.

We adjust the alpha-level of the correlations’ inferential tests using the Bonferroni correction in order to control for the number of erroneous theoretical inferences. Each kind of validity is taken as an independent theoretical claim following the intuition that the assessed musical skill measure might well be valid on one dimension (e.g., discriminant validity) but not another (e.g., criterion validity). All tables and Fig 2 report uncorrected *p*-values while the text reports both uncorrected and corrected *p*-values. The latter correct for three (criterion validity), or two (discriminant and convergent validity) comparisons.

## Results

### Test structure

In Table 1 we show the pattern of associations of the overall brief PROMS score and its subtests with each other. Each of the subtests correlates very highly with the overall brief PROMS score (all *r*s > .77). Amongst each other, the subtests correlate between *r* = .46 and *r* = .62, i.e. the effect size of the association is mostly large (*r* > .5) according to Cohen’s criteria [18].

**Table 1:** Pearson product moment correlations of the brief PROMS and its subtests with each other. Sample size is given in brackets.

	melody	tuning	tempo	rhythmic accent
brief PROMS total	*r*_(157)_ = .812***	*r*_(157)_ = .833***	*r*_(157)_ = .776***	*r*_(157)_ = .841***
melody		*r*_(160)_ = .560***	*r*_(157)_ = .458***	*r*_(157)_ = .619***
tuning			*r*_(157)_ = .559***	*r*_(157)_ = .580***
tempo				*r*_(157)_ = .552***

Note. The maximal absolute difference between the Pearson *r* values reported here and their associated Spearman *rho* values is .038 units.

***p_uncorrected_ < .001.

### Validity measures

Table 2 shows how the different validity measures correlate with each other. This assessment is of course only possible if the same participants provide information for different measures. This is not the case for all combinations of validity measures, see Table 2 (*N* = 0).

**Table 2:** Associations among the validity measures. Sample size is given in brackets.

	backward digit span	musical training years	musicianship status	self-rated musical talent	musical task accuracy (full attention)	musical task accuracy (divided attention)
forward digit span	*r*_(101)_ = .326**	*r*_(101)_ = .103	*r*_(100)_ = .078	*r*_(101)_ = .135	*N* = 0	*r*_(57)_ = .148
backward digit span		*r*_(101)_ = .016	*r*_(100)_ = -.096	*r*_(101)_ = .003	*N* = 0	*r*_(57)_ = .252†
musical training years			*r*_(160)_ = .647***	*r*_(161)_ = .644***	*r*_(60)_ = .156	*r*_(57)_ = .366**
musicianship status				*r*_(160)_ = .806***	*r*_(60)_ = .212	*r*_(57)_ = .419**
self-rated musical talent					*r*_(60)_ = .230†	*r*_(57)_ = .420**
musical task accuracy (full attention)						*N* = 0

Note. The maximal absolute difference between the Pearson *r* values reported here and their associated Spearman *rho* values is .082 units.

† .05 < *p*_uncorrected_ < .1

**p_uncorrected_ < .01

***p_uncorrected_ < .001.

The two discriminant validity measures (forward and backward digit span) are merely moderately correlated (*r* = .33), justifying a distinction between short term memory (forward digit span) and working memory (backward digit span). The criterion validity measures (musical training years, musicianship status, self-rated musical talent), on the other hand, are strongly correlated with each other (*r*s > .64).

Furthermore, it is noteworthy that the correlations of musical task accuracy under *divided* attention with musical skill measures are somewhat greater (*r*s > .36) than the correlations of musical task accuracy under *full* attention with musical skill measures (*r*s < .24). This could reflect the different trial counts available for the two different musical skill measures. A measure with more observations (100 trials under divided attention) is probably less noisy and can therefore display a higher correlation with another variable than a measure with less observations (40 trials under full attention). Alternatively, musical training might affect attention abilities which in turn impact harmony perception. According to this speculative account, the influence of musical training on harmony perception is increased when attention is taxed because attention is differently developed for participants with different amounts of musical training.

### Discriminant validity

Discriminant validity assesses whether tests measuring unrelated concepts do not correlate with each other. As can be seen in Table 3 and Fig 2A, the brief PROMS does not correlate with backward digit span (*r* = .04, *p*_uncorrected_ = .71, *p*_corrected_ > 1), neither do any PROMS subtests individually (*r*s ≤ .100). Even though the correlation of the PROMS total score with forward digit span reaches significance (*r* = .22, *p*_uncorrected_ = .03, *p*_corrected_ = .06, see Fig 2B), the association is weaker than for other music ability tests. These exhibit Pearson correlation coefficient values of *r* ≥ .4 [3,8], i.e. beyond the 95% Confidence Interval of the association we find here (95% CI = .02–.40). When looking at the four sub-tests separately (see Table 3), it becomes clear that the tuning (*r* = .05) and tempo subtests (*r* = .10) prevent the overall brief PROMS score from correlating strongly with forward digit span. Overall, the brief PROMS shows surprisingly good discriminant validity with short term memory and working memory.

**Table 3:** Pearson product moment correlations of the brief PROMS and its subtests with measures of validity. Sample size is given in brackets.

	brief PROMS total	melody	tuning	tempo	rhythmic accent
forward digit span	*r*_(97)_ = .223*	*r*_(100)_ = .238*	*r*_(100)_ = .053	*r*_(97)_ = .104	*r*_(97)_ = .285**
backward digit span	*r*_(97)_ = .039	*r*_(100)_ = .100	*r*_(100)_ = —.082	*r*_(97)_ = .005	*r*_(97)_ = .092
musical training years	*r*_(157)_ = .450***	*r*_(160)_ = .482***	*r*_(160)_ = .416***	*r*_(157)_ = .201*	*r*_(157)_ = .357***
musicianship status	*r*_(156)_ = .475***	*r*_(159)_ = .456***	*r*_(159)_ = .446***	*r*_(156)_ = .289***	*r*_(156)_ = .354***
self-rated musical talent	*r*_(157)_ = .513***	*r*_(160)_ = .527***	*r*_(160)_ = .404***	*r*_(157)_ = .372***	*r*_(157)_ = .366***
musical task accuracy (full attention)	*r*_(60)_ = .364**	*r*_(60)_ = .340**	*r*_(60)_ = .425**	*r*_(60)_ = .094	*r*_(60)_ = .315*
musical task accuracy (divided attention)	*r*_(53)_ = .398**	*r*_(56)_ = .347**	*r*_(56)_ = .421**	*r*_(53)_ = .274*	*r*_(53)_ = .288**

Note. The maximal absolute difference between the Pearson *r* values reported here and their associated Spearman *rho* values is .065 units.

*p_uncorrected_ < .05

**p_uncorrected_ < .01

***p_uncorrected_ < .001.

### Criterion validity

Criterion validity assesses a test’s association with concrete criteria outside the lab, e.g. teacher assessments or attainments in music exams. We have no access to such data and therefore use three proxies instead. Following Law & Zentner [2], these are years of musical training and musicianship status (coded: nonmusician [= 1]; music-loving nonmusician [= 2]; amateur musician [= 3]; semiprofessional musician [= 4]; professional musician [= 5]). The third measure is self-rated musical talent (10-point Likert scale; not at all talented [= 0] to extremely talented [= 10]).

Just like Law and Zentner [2], we find a correlation between the brief PROMS and years of formal musical training (*r* = .45, *p*_uncorrected_ < .001, *p*_corrected_ < .001) as well as between the brief PROMS and musicianship status (*r* = .47, *p*_uncorrected_ < .001, *p*_corrected_ < .001), see Table 3, Fig 2C and 2D. The observed association strength is remarkably similar to that reported by Law and Zentner [2] for the correlation between the brief PROMS and years of formal musical training: *r*_(39)_ = .39. Going beyond associations with measures of musical training, we investigate the relation to self-rated musical talent, see Fig 2E. The moderate association strength (*r* = .51, *p*_uncorrected_ < .001, *p*_corrected_ < .001) suggests that the brief PROMS partly measures musical talent as understood by relatively untrained participants. All four subtests show association strengths which are not much lower (*r*s > .36). Overall, this analysis confirms the comparatively good criterion validity of the brief PROMS.

### Convergent validity

If two tasks which measure the same construct correlate, they exhibit convergent validity. The brief PROMS correlates well with our measure of musical task accuracy which is based on harmonic closure ratings either acquired in a full attention setting (*r* = .36, *p*_uncorrected_ = .004, *p*_corrected_ = .009) or in a divided attention setting (*r* = .40, *p*_uncorrected_ = .003, *p*_corrected_ = .006), see Table 3, Fig 2F and 2G.

In order to check whether the correlation between musical task accuracy and the brief PROMS is due to a third variable influencing both closure ratings and the brief PROMS, we include the self-reported musical skill measures in a partial correlation analysis. Given that musical training years, musicianship status, and self-rated musical talent are highly correlated (see Table 2), we summarize them in a single principal component which in this case accounts for 80% of the variance and is correlated with the brief PROMS (*r* = .53, *p* < .001, see Fig 2H). In the case of musical task accuracy under full attention, the correlation with the brief PROMS score is still significant when self-reported musical skill measures are held constant through a partial correlation (*r*_partial_ = .30, *p*_uncorrected_ = .019, *p*_corrected_ = .038). However, the correlation between musical task accuracy under *divided* attention and the brief PROMS score is no longer significant after holding self-reported musical skill measures constant (*r*_partial_ = .17, *p*_uncorrected_ = .232, *p*_corrected_ = .465). Overall, these findings suggest that the brief PROMS truly measures musical skills rather than just the ability to compare two auditory stimuli. However, whether this result is a reflection of musical training, talent and musicianship status influencing both the brief PROMS and music harmony perception skills is ambiguous.

### Construct validity

At the suggestion of an anonymous reviewer, we also include a validity measure combining all previously reported measures of validity. Westen and Rosenthal [19] propose *r*_alerting-CV_ and *r*_contrast-CV_ as measures of general construct validity. Given that not all participants contributed to all validity measures, only *r*_alerting-CV_ can be calculated here. It is a measure of fit between the predictions of discriminant, criterion, and convergent validity, and the observed values. It is not associated with a *p*-value.

The ideal pattern of correlations between validity measures and the brief PROMS is shown in Table 4‘s first column. We assume that discriminant validity measures should not be correlated with the brief PROMS (*r* = 0) while criterion and convergent validity measures should correlate as highly as possible (given the test-retest reliability of the brief PROMS at *r* = .84). The resulting *r*_alerting-CV_ of .90 (the correlation between the predicted values in column 2 of Table 4 and the observed values in column 4) points to an overall very good fit between the ideal pattern of construct validity and the observed pattern. None of the subtests alone displays a poor fit either: melody (*r*_alerting-CV_ = .84), tuning (*r*_alerting-CV_ = .98), tempo (*r*_alerting-CV_ = .71), and rhythmic accent (*r*_alerting-CV_ = .76).

**Table 4:** Values required for the calculation of the construct validity measure *r*_alerting-CV_.

	predicted correlation (*r*) with brief PROMS	Fischer *Z* of predicted *r* (demeaned)	observed *r*	Fischer *Z* of observed *r*
forward digit span	*r* = 0	*Z*_*r_demeaned*_ = -0.872	*r* = .223	*Z*_*r*_ = 0.227
backward digit span	*r* = 0	*Z*_*r_demeaned*_ = -0.872	*r* = .039	*Z*_*r*_ = 0.039
musical training years	*r* = .84	*Z*_*r_demeaned*_ = 0.349	*r* = .450	*Z*_*r*_ = 0.485
musicianship status	*r* = .84	*Z*_*r_demeaned*_ = 0.349	*r* = .475	*Z*_*r*_ = 0.516
self-rated musical talent	*r* = .84	*Z*_*r_demeaned*_ = 0.349	*r* = .513	*Z*_*r*_ = 0.567
musical task accuracy (full attention)	*r* = .84	*Z*_*r_demeaned*_ = 0.349	*r* = .364	*Z*_*r*_ = 0.381
musical task accuracy (divided attention)	*r* = .84	*Z*_*r_demeaned*_ = 0.349	*r* = .398	*Z*_*r*_ = 0.421

Note. Predicted correlations between validity measures and the brief PROMS are zero for discriminant validity measures. They are the same as the test-retest validity of the brief PROMS for the criterion and convergent validity measures, following the intuition that no measure can better predict brief PROMS scores than the brief PROMS itself.

## Discussion

There is no agreement on how to measure musical aptitude even though it provides interesting avenues for research. Newly developed measures of musical skills should be rigorously, psychometrically assessed before being widely applied, as we do here for the brief PROMS [2]. For this evaluation we focus on various measures of validity and show that on all of them the chosen measure of musical perception skills performs well. In terms of discriminant validity, its correlations with short term memory and working memory are low despite the nature of the task which asks participants to hold a stimulus in mind in order to compare it to a second stimulus. Perhaps unsurprisingly, the PROMS subtests with longer stimuli (melody and rhythmic accent) display somewhat stronger (but still weak) correlations with short term memory than the subtests with shorter stimuli (tuning, tempo).

As opposed to the weak correlations with short term memory and working memory, the associations with musical training, musicianship status, and self-rated musical talent are high. This suggests good criterion validity, meaning that the brief PROMS is associated with ‘real world’ measures of the same concept. The observed correlations might not be even higher because of the presence of musical sleepers (musically untrained people with great music perception skills, see Fig 2C or 2H top left corner) and sleeping musicians (musically trained people with surprisingly poor music perception skills, see Fig 2C or 2H bottom right corner) [2].

Furthermore, in two independent samples, brief PROMS scores correlate well with a different kind of music measure based on closure ratings of harmonic sequences. We take this as a sign of good convergent validity—the brief PROMS measures an underlying concept (musical perception skill) also measured by the closure rating task. This result is surprising because no PROMS subtest actually requires any harmonic understanding, suggesting that the brief PROMS captures a general form of musical aptitude which generalizes to unrelated tasks, as hypothesized by Law and Zentner [2]. The pattern of correlations of the subtests suggests that next to general musical aptitude there are also music perception subskills which can be more or less developed in the same person. Specifically, the harmonic judgment accuracy measure correlates well with the only subtest including chords (tuning subtest) but weakly with timing-related subtests (tempo, rhythmic accent). This suggests that the brief PROMS can, at least to some degree, measure a profile of strengths and weaknesses in music perception skills. It remains ambiguous whether this pattern of association between the brief PROMS and the harmony perception task is due to both these tasks being affected by musical training or whether the association between the brief PROMS and harmony perception goes beyond musical training. A partial correlation analysis reveals contradictory findings for two independent samples. Future research might elucidate this point.

Finally, an overall construct validity analysis [19], which combines all aforementioned patterns of association between the brief PROMS and validity measures, suggests that the brief PROMS conforms remarkably well to an ideal pattern of discriminant, criterion and convergent validity. None of the individual subtests performs poorly either. One can conclude that the brief PROMS shows good overall construct validity.

One of the PROMS subtests, the tuning subtest, which asks participants whether two chords are tuned in the same way, is noteworthy. It shows a remarkably good pattern of discriminant, convergent, criterion, and overall construct validity. It is not associated with working memory and still correlates significantly with musical training years, musicianship status, self-rated musical talent and musical task accuracy. This subtest’s unusual performance might not just result from the short stimuli. One could speculate that by asking for a tuning judgment, participants could compare each chord to a tuning standard held in long term memory. As a result, they do not really compare the standard with the comparison stimulus, but instead simply classify each as well tuned or mistuned. With this strategy a matching classification (e.g., both chords well tuned) suggests matching standard and comparison stimuli. If this hypothesis is true, the tuning subtest depends on a culturally specific representation of tuning held in long term memory and shared among listeners of Western music. This suggests that such a subtest is a valuable part of any evaluation of music ability in Western listeners but perhaps not in non-Western listeners.

Does the promising outcome of the psychometric evaluation of the brief PROMS which we present here mean music cognition researchers should adopt this measure? We believe that the answer depends on the research question. Different musical aptitude measures offer the interested researcher different advantages. We have shown that one advantage of the brief PROMS is its very good pattern of discriminant, convergent and criterion validity. However, other considerations could also play a role. For example, some measures of musical skills are claimed to be better suited for younger [5] or musically impaired samples [1]. While others require shorter testing times of less than 20 minutes [6,8]. Yet others claim to measure a somewhat broader concept of musical sophistication which goes beyond music skills trained in formal instrument lessons [9,11]. Moreover, if the aim is to compare any results to previous studies using classical musical aptitude tests, the interested researcher is probably well advised to opt for these classical tests instead [20,21]. We hope that the results presented here help in determining which music ability measure to choose. We believe that the brief PROMS should be on the list of musical skill tests to consider.

## Supporting Information

S1 File PROMS_analyser.R. Analysis code for recreating all analyses and figures in this manuscript.
(R) Click here for additional data file.

S2 File PROMS_MASTER.csv. Subject specific information reported in this manuscript.
(CSV)

S3 File Exp1_subj_items.csv. Required for the calculation of Cronbach’s alpha for the musical task accuracy measure.
(CSV)

S4 File post-test_subj_items.csv. Required for the calculation of Cronbach’s alpha for the musical task accuracy measure.
(CSV)

## References

[1] Peretz I, Champod AS, Hyde K. Varieties of musical disorders: the Montreal battery of evaluation of amusia. Annals of the New York Academy of Sciences. 2003;999: 58–75. 10.1196/annals.1284.006 14681118

[2] Law LNC, Zentner M. Assessing musical abilities objectively: Construction and validation of the Profile of Music Perception Skills. PLoS ONE. 2012;7: e52508 10.1371/journal.pone.0052508 23285071 PMC3532219

[3] Anvari SH, Trainor LJ, Woodside J, Levy BA. Relations among musical skills, phonological processing, and early reading ability in preschool children. Journal of Experimental Child Psychology. 2002;83: 111–130. 10.1016/S0022-0965(02)00124-8 12408958

[4] Slevc LR, Miyake A. Individual differences in second-language proficiency: Does musical ability matter? Psychol Sci. 2006;17: 675–681. 16913949 10.1111/j.1467-9280.2006.01765.x

[5] Peretz I, Gosselin N, Nan Y, Caron-Caplette E, Trehub SE, Béland R. A novel tool for evaluating children’s musical abilities across age and culture. Frontiers in Systems Neuroscience. 2013;7: 30 10.3389/fnsys.2013.00030 23847479 PMC3707384

[6] Ullén F, Mosing MA, Holm L, Eriksson H, Madison G. Psychometric properties and heritability of a new online test for musicality, the Swedish Musical Discrimination Test. Personality and Individual Differences. 2014;63: 87–93. 10.1016/j.paid.2014.01.057

[7] Gingras B, Honing H, Peretz I, Trainor LJ, Fisher SE. Defining the biological bases of individual differences in musicality. Phil Trans R Soc B. 2015;370: 20140092 10.1098/rstb.2014.0092 25646515 PMC4321133

[8] Wallentin M, Nielsen AH, Friis-Olivarius M, Vuust C, Vuust P. The Musical Ear Test, a new reliable test for measuring musical competence. Learning and Individual Differences. 2010;20: 188–196. 10.1016/j.lindif.2010.02.004

[9] Schaal NK, Bauer A-KR, Müllensiefen D. Der Gold-MSI: Replikation und Validierung eines Fragebogeninstrumentes zur Messung Musikalischer Erfahrenheit anhand einer deutschen Stichprobe. Musicae Scientiae. 2014;18: 423–447. 10.1177/1029864914541851

[10] Pasinski AC, Hannon EE, Snyder JS. How musical are music video game players? Psychon Bull Rev. 2016; 1–6. 10.3758/s13423-015-0998-x 26732385

[11] Müllensiefen D, Gingras B, Musil J, Stewart L. The Musicality of Non-Musicians: An Index for Assessing Musical Sophistication in the General Population. PLOS ONE. 2014;9: e89642 10.1371/journal.pone.0089642 24586929 PMC3935919

[12] Brown S, Jordania J. Universals in the world’s musics. Psychology of Music. 2013;41: 229–248. 10.1177/0305735611425896

[13] Ericsson AK, Krampe RT, Tesch-Römer C. The role of deliberate practice in the acquisition of expert performance. Psychological Review. 1993;100: 363–406. 10.1037/0033-295X.100.3.363

[14] Macnamara BN, Hambrick DZ, Oswald FL. Deliberate Practice and Performance in Music, Games, Sports, Education, and Professions A Meta-Analysis. Psychological Science. 2014;25: 1608–1618. 10.1177/0956797614535810 24986855

[15] Hansen M, Wallentin M, Vuust P. Working memory and musical competence of musicians and non-musicians. Psychology of Music. 2013;41: 779–793. 10.1177/0305735612452186

[16] Kunert R, Willems RM, Hagoort P. Language influences music harmony perception: effects of shared syntactic integration resources beyond attention. Royal Society Open Science. 2016;3: 150685 10.1098/rsos.150685 26998339 PMC4785990

[17] Groth-Marnat G. The Wechsler intelligence scales. Specific Learning disabilities and difficulties in children and adolescent Psychological assessment and evaluation. 2001; 29–51.

[18] Cohen J. A power primer. Psychological bulletin. 1992;112: 155 19565683 10.1037//0033-2909.112.1.155

[19] Westen D, Rosenthal R. Quantifying construct validity: Two simple measures. Journal of Personality and Social Psychology. 2003;84: 608–618. 10.1037/0022-3514.84.3.608 12635920

[20] Seashore CE, Lewis D, Saetveit JG. Measures of Musical Talents (test) Psychological Corporation; 1960.

[21] Gordon EE. Advanced measures of music audiation: kit. CD. Advanced measures of music audiation. GIA Publ.; 1989.

